# Supplementary material for: Serum miRNAs associated with tumor-promoting cytokines in non-small cell lung cancer
Source: PLoS One. 2020 Oct 30;15(10):e0241593. doi: 10.1371/journal.pone.0241593 (PMC7598461; doi:10.1371/journal.pone.0241593)
Supplement: S1 Table — (PDF) [file pone.0241593.s001.pdf]

Supplementary Table 1

List of the genes associated with the consistently up-regulated miRNA

miRNA	Potential total genes	Validated targets*	Gene name of validated targets*
miR-145	225	135	BNIP3, CDKN1A, STAT1, YES1, FSCN1, MYC, KLF5, SOX2, KLF4, MUC1, MYO6, ITGB8, IRS1, HOXA9, FLI1, DFFA, IGF1R, SRGAP1, SWAP70 , JAG1, ADAM17 , RTKN, SOX9, SMAD3, TGFBR2, CTNND1, CDK6, ARF6, HMGA2, E2F3, VEGFA, POU5F1, ROBO2, EIF4E, CDK4, SERPINE1, NEDD9, PAK4, DDX17, ERG, NRAS, ILK, EPAS1, ETS1, RREB1, CD44,BRAF, SP1,TNFSF13, DDX6, ADD3, SP7, HDAC11, SENP1, NAIP, CBFB, PPP3CA, CLINT1, PARP8, TMOD3, EGFR, MAP3K3, IFNB1, TIRAP, KRT7, PPM1D, MYRF, CPEB4, FZD7, IRS2, ESR1, NUDT1, CTGF, SOCS7, MDM2, CDH2, HDAC2, F11R, ARL6IP5, AKR1B10, C11orf65, HLTF, GMFB, SERINC5, MEST,ALPPL2, NDRG2, DTD1, TPM3, MAP2K6, CEP19, TPRG1, GOLM1, CCDC43, MMP1, PTP4A2, TMEM9B, MMP12,MTMR14, ALDH3A1, NDUFA4, FAM3C, LYPLA2, FAM45A, PIGF, AP1G1, PHF17, NIPSNAP1, KREMEN1, MMP14, ABRACL, MIXL1, TSPAN6, PODXL, APH1A, ABHD17C, NANOG, MYO5A, COL5A1, ACTB, PADI1, PHACTR2, SNX24, KIF21A, SAMD5, AIM1, DDC, ANGPT2, ROCK1, RPS6KB1, CD28, NFATC1, CFTR, ABCC1, SPTBN1
miR-320a	562	31	Polr3d, POLR3D, TAC1, TFRC, MCL1, HSPB6, AQP1, AQP4, NPR1, MAPK1, IGF1R, VDAC1, ITGB3, GNAI1, RAC1, PDCD4, BMI1, ARF1, PBX3, NRP1, NFATC3, TRPC5, PTEN, BANP, RAB14, SUZ12, FOXM1, KITLG, RUNX2, MTDH, YWHAZ
miR-151a-3p	75	6	ZNF763, NTRK3, CCNE1, ZNF763, NTRK3, TWIST1
miR-16	1531	66	BMI1, HMGA1, ACVR2A, PDCD4, RAB21, WT1, CDK6, CCND3, CCND1, CCNE1, RARS, PURA, PTGS2, ITGA2, FGF2, ARL2, BCL2, CCNT2, BRCA1, AKT3, WNT3A, VEGFA, SKAP2, CADM1, TPPP3, MYB, AURKB, CAPRIN1, PPM1D, CHUK, TP53, ZYX, NCOR2, Ccnd1, AXIN2, KDR, FGFR1, HMGA2, PIM1, IFNG, UNG, RECK, RPS6KB1, MTOR, PRDM4, MAP7, APP, WEE1, CDS2, SOX6, IGF1R, RAF1, KRAS, SLC6A4, YAP1, SOX5, BIRC5, CHEK1, RICTOR, BDNF, HDGF, WNT4, Tnf, BACE1, NCSTN, OPRM1
miR-200b-3p	162	70	PTPN12, ZEB2, BAP1, ZEB1, RERE, Zeb2, Zeb1, ETS1, GATA4, FN1, WASF3, ZFPM2, MATR3, RNF2, BMI1, E2F3, VEGFA, FLT1, KDR, RND3, CCNE2, BCL2, XIAP, SMAD2, CREB1, KLHL20, ELMO2, PTPRD, ERBB2IP, WDR37, TCF7L1, VAC14, HOXB5, RIN2, RASSF2, KLF11, SEPT7, SHC1, MYB, QRSL1, SUZ12, WNT1, DNMT3A, DNMT3B, SP1, MSN, DNMT1, EZH2, HFE, DLC1, HNRNPA3, FERMT2, RAB21, RAB3B, RAB18, RAB23, OXR1, FOXG1, NOTCH1, BTC, CDKN1B, LOX, PHLPP1, SEC23A, ROCK2, PIN1, DDX53, JUN, MAPK7, RHOA

miRNA	Potential total genes	Validated targets*	Gene name of validated targets*
miR-205	172	42	LRP1, ZEB1, DDX5, INPPL1, MED1, E2F5, E2F1, ERBB3, ZEB2, PRKCE, VEGFA, SIGMAR1, IL24, IL32, EGLN2, TP73, CYR61, CTGF, ERBB2, LAMC1, LRRK2, YES1, SRC, BCL2, SMAD1, SMAD4, YY1, PTPRM, AR, BCL6, ACSL4, ITGA5, ACSL1, HMGB3, PTEN, ESRRG, PHLPP2, KCNJ10, EZR, LMNA, RUNX2, HMGB1
miR-574	351	3	Dera, Nusap1, FOXN3
miR-125b	443	96	BMPR1B, EIF4EBP1, HMGA2, HMGA1, GLI1, NKIRAS2, SMO, TP53, LIF, VDR, SGPL1, BAK1, ERBB3, ERBB2, BMF, KLF13, NTRK3, LIN28A, CBFB, AKT1, CYP24A1, RAF1, PRDM1, IRF4, GRIN2A, CDKN2A, KRT7, TP53INP1, LACTB, STAT3, E2F3, IGF2, LIN28B, Bak1, BBC3, Tef, PPP1CA, PRKRA, BCL2, ETS1, RPS6KA1, TNFAIP3, PIGF, BCL3, TBC1D1, DGAT1, FGFR2, ARID3B, SMAD4, MCL1, IL6R, STARD13, ABTB1, HK2, E2F2, MMP13, MAPK14, EPO, MUC1, NES, CDH5, ARID3A, BCL2L2, NCOR2, Prtg, EIF5A2, MXD1, PIAS3, PIK3CD, PCTP, LIPA, GSS, IKZF2, IKZF3, IKZF4, ICAM2, VPS4B, BTG2, SET, CCNJ, ENPEP, CSNK2A1, MEGF9, MAN1B1, EPOR, AHRR, SCNN1A, VPS51, SIRT7, DUSP6, TET2, SPHK1, MMP2, MMP26, MAP3K11, SFRP5
miR-186	731	9	FOXO1, P2RX7, AKAP12, CSNK2A1, PTTG1, TERF2IP, NCSTN, ABCB1, SETD2
miR-18a	257	34	ESR1, PTEN, CTGF, NCOA3, TNFSF11, NR3C1, TSC22D3, HIF1A, TGFBR2, Prmt5, Myc, SMAD4, HSF2, ATM, NEDD9, CDK19, DICER1, SMAD3, CREBL2, PHLPP1, PIAS3, BCL2, SMAD2, SDC4, STK4, BCL2L10, FCGR2B, NEO1, FAS, TP53, DNMT1, IRF2, RUNX1, MEF2D

* Targeted genes that are supported by strong and experimental evidences from the miRTarBase 7.0
