# Supplementary material for: Serum miRNAs associated with tumor-promoting cytokines in non-small cell lung cancer
Source: PLoS One. 2020 Oct 30;15(10):e0241593. doi: 10.1371/journal.pone.0241593 (PMC7598461; doi:10.1371/journal.pone.0241593)
Supplement: S2 Table — (PDF) [file pone.0241593.s002.pdf]

Supplementary Table 2

List of the genes associated with the consistently down-regulated miRNA

miRNA	Potential total genes	Validated targets*	Gene name of validated targets*
miR-1285-3p	180	2	TP53, TGM2
miR-1243	37	0	No data
miR-661	329	9	MTA1, STARD10, VCL, MTA2, PVRL1, MCL1, MDM4, MDM2, MGMT
miR-708	117	22	BMI1, ZEB2, BIRC5, MPL, N4BP1, OTUB1, TEX261, AKT2, CD44, TMEM88, EYA3, NNAT, AKT1, CCND1, MMP2, EZH2, PARP1, BCL2, CASP2, CD274, CNTFR, SMAD3
miR-572	17	1	CDKN1A
miR-206	96	32	MET, NOTCH, ESR1, UTRN, FSTL1, TAC1, PAX3, Tppp, CCND2, GJA1, CDK4, ACTL6A, NR1H3, BCL2, NRP1, GPD2, TKT, PGD, G6PD, VAMP2, SMARCB1, KRAS, BDNF, SFRP1, FRS2, IGF1R, KLF4, HDAC4, STC2, CCND1, DUX4L9, ANXA2
let-7d	365	9	HMGA2, APP, DICER1, SLC11A2, PDGFA, IL13, MPL, EIF2C1, TNFRSF10B
miR-15a	687	47	BMI1, WNT3A, MYB, CDC25A, CCND2, PDCD4, RAB21, BCL2, WT1, CCND1, CCNE1, BACE1, DMTF1, BRCA1, AKT3, SKAP2, CADM1, TMEM184B, APP, UCP2, VEGFA, TSPYL2, NFKB1, CHUK, TP53, FGF7, CLCN3, CRKL, MN1, Ccnd1, HMGA1, HMGA2, IFNG, PURA, RECK, DLK1, FOXO1, REPIN1, KLF4, YAP1, CARM1, SOX5, HSPA1B, KLF6, PHLPP1, RET, CXCL10

* Targeted genes that are supported by strong and experimental evidences from the miRTarBase 7.0
