# Supplementary material for: Serum miRNAs associated with tumor-promoting cytokines in non-small cell lung cancer
Source: PLoS One. 2020 Oct 30;15(10):e0241593. doi: 10.1371/journal.pone.0241593 (PMC7598461; doi:10.1371/journal.pone.0241593)
Supplement: S3 Table — (PDF) [file pone.0241593.s003.pdf]

Supplementary Table 3

List of the genes associated with the inconsistency reported miRNA

miRNA	Potential total genes	Validated targets*	Gene name of validated targets*
miR-20a	1037	65	HIF1A, TCEAL1, CCND1, E2F1, BMPR2, CDKN1A, TGFBR2, MAP3K12, BCL2, MEF2D, PTEN, APP, RUNX1, NRAS, VEGFA, MUC17, MYC, BNIP2, THBS1, CCND2, E2F3, MAPK9, RB1, RBL1, RBL2, WEE1, IRF2, KIT, EGLN3, PPARG, BAMBI, CRIM1, MAP2K3, PURA, ARHGAP12, TSG101, SIRPA, UBE2C, STAT3, LIMK1, PHLPP2, GJA1, DUSP2, ITGB8, SMAD7, MAP3K5, MCL1, TP53INP1, EGR2, ABL2, ATG16L1, ANKH, PRRG1, PRKG1, RGS5, ETV1, EPAS1, FBXO31, TP53, DNMT1, PKD1, PKNOX1, RB1CC1, NFKBIB, KIF26B
miR-25-3p	442	26	PRMT5, BCL2L11, KLF4, CDKN1C, KAT2B, TP53, WDR4, CDH1, CCL26, MDM2, PTEN, EZH2, SMAD7, MAP2K4, DSC2, ATP2A2, RECK, ERBB2, TCEAL1, LATS2, REV3L, NOX4, FBXW7, HAND2, MYC, CPEB1
miR-223	255	0	No data
let-7f	370	23	KLK10, KLK6, PRDM1, IL13, MPL, CYP19A1, COPS8, NKX2-1, SLC5A5, MYC, GPS1, TG, CCND1, COPS6, CDKN1A, MYH9, SOCS3, ELF4, DYRK2, CCL7, EIF2C1, HDAC2, IL6
miR-20b	886	21	STAT3, HIF1A, CDKN1A, HIPK3, MYLIP, PPARG, CRIM1, ARID4B, BAMBI, ESR1, VEGFA, MUC17, EFNB2, EPHB4, MAPK9, LIMK1, PTEN, CCND1, KLF6, BRCA1, RB1CC1
let-7a	605	61	CDK6, MYC, BCL2, NKIRAS2, ITGB3, NF2, NRAS, KRAS, PRDM1, lin-41, TRIM71, FOXA1, NR1I2, VDR, RAVER2, HMGA2, HMGA1, EIF2C4, trim71, APP, E2F1, wech, UHRF2, DICER1, HRAS, IGF2, LIN28A, NFKB1, ZFP36L1, THBS1, TUSC2, SLC20A1, NEFM, MEIS1, EGR3, HNRPDL, AMMECR1, CASP3, CASP8, CASP9, IL6, E2F2, CCND2, IGF2BP1, CDC34, EWSR1, MPL, CCR7, TMED7, CDKN1A, HAS2, EGFR, RRM2, AGO1, EZH2, UHRF1, PAK1, AURKB, RAB40C, ARG2, TNFRSF10B
miR-17	1151	79	ZNFX1, CCL1, GPR137B, NABP1, NPAT, YES1, JAK1, PTEN, CDKN1A, PTPRO, PKD2, BCL2L11, E2F1, MAP3K12, BCL2, MEF2D, RUNX1, APP, VEGFA, MAPK9, DNAJC27, FBXO31, HIF1A, TGFBR2, TNFSF12, MUC17, BMPR2, CCND1, MYC, NCOA3, THBS1, SMAD4, ICAM1, SELE, CCND2, E2F3, RB1, RBL1, RBL2, WEE1, RND3, SMURF1, TCF3, TCEAL1, HSPB2, MMP2, HBP1, SIRPA, UBE2C, PHLPP1, Jak2, Stat5a, Bcl2, TP53INP1, ITGB8, BMP2, SOCS6, LIMK1, KAT2B, TIMP3, ZBTB4, PDLIM7, MAPK14, STAT3, NPAS3, LDLR, CREB1, PRRG1, TGFB1, CLU, ADAR, MDM2, ETV1, EPAS1, TBC1D2, FAS, TP53, DNMT1, PKNOX1

* Targeted genes that are supported by strong and experimental evidences from the miRTarBase 7.0
